# P40 The effectiveness of contextualizing multimodal strategy on doctors to improve hand hygiene compliance in surgical wards at Ahmadu Bello University Teaching Hospital Zaria, Nigeria

**DOI:** 10.1093/jacamr/dlad066.044

**Published:** 2023-06-14

**Authors:** Yahaya Yaqub, Zainab Lamido Tanko, Yahya Umar Uthman, Aliyu Aminu, Joan Ejembi, Kath Houston

**Affiliations:** Ahmadu Bello University Zaria, Nigeria; Kaduna State University, Nigeria; Bayero University Kano, Nigeria; Bayero University Kano, Nigeria; Ahmadu Bello University Zaria, Nigeria; Lancaster University, Lancaster, UK

## Abstract

**Background:**

Hospital acquired infection (HAI) is a major problem in healthcare delivery worldwide despite medical advances. A low HH compliance rate of 17.1% was found among surgical health workers at Ahmadu Bello University Teaching Hospital (ABUTH) Zaria, Nigeria. Although hand hygiene (HH) is a proven low-cost means to curtail HAI, low HH compliance is a global reality. The WHO multimodal strategy attempts to address low HH compliance within the frame of contextual realities.

**Methods:**

This was an interventional study utilizing action research through a mixed method approach to investigate the effectiveness of employing a contextualized multimodal strategy to enhance the HH compliance rate of doctors at ABUTH Zaria. The study was from August to October 2022. A behavioural change campaign (BCC) HH workshop was carried out on doctors, and data collection followed immediately in the surgical wards (i.e. clusters) that had received environmental modification through the provision HH posters and nurses for visual and verbal reminders, respectively. The four clusters were: visual reminders only, verbal reminders only, visual and verbal reminders combined and neither visual nor verbal reminders (i.e. control). All clusters received an uninterrupted supply of alcohol-based hand rubs (ABHR) throughout the study period. The HH observation proforma for ‘five moments of HH’ template was used to collect the quantitative data through direct observation while qualitative data were by one-on-one interviews on doctors in surgical wards at ABUTH Zaria.

**Results:**

The cumulative HH compliance rate by doctors was 69% (*N* = 1774). This was significantly different from the baseline HH compliance rate of 17.1% before this study’s intervention (*P* < 0.0001, C. I. 45.5% to 57.7%). HH was observed from doctors most in wards with both visual and verbal reminders (78%) while lowest (59%) where visual and verbal reminders were not provided, i.e. C4 (*n* = 444). Compared with the baseline HH compliance, all interventions were significant. However, compared with C4, visual reminders only (C1) had insignificant effect on influencing the HH practice of doctors while visual and verbal reminders combined. Verbal reminders only (C2) were found to be as effective as when combined with visual reminders (78%) as the difference was statistically insignificant (*P* = 0.4791, CI −3.5% to 7.5%, χ^2^ = 0.5). However, all doctors reported being motivated to perform HH by the presence of ABHR which was similar to the significance found even in the ward where no verbal or visual reminders (59%, *n* = 444) were used compared with the baseline (17%, *n* = 286) before this intervention study (*P* < 0.0001, CI 35% to 48%, χ^2^ = 125). All respondents reported hearing about the HH behavioural change workshop even though only one attended.

**Conclusions:**

Continuous workshop on HH is necessary to sustain awareness of health workers on the importance of hand hygiene. The presence of ABHRs alone was enough motivation for doctors to perform HH but verbal reminders were significant in sustaining this positive attitude towards HH. A scaling of this intervention is needed in other departments so that staff can have changes made to their values towards healthcare delivery as well at ABUTH Zaria. Contextualizing the WHO multimodal strategy is an efficient and acceptable means of addressing the problem of low HH compliance.

